# Bioactivity of Isostructural Hydrogen Bonding Frameworks Built from Pipemidic Acid Metal Complexes

**DOI:** 10.3390/molecules25102374

**Published:** 2020-05-20

**Authors:** Paula C. Alves, Patrícia Rijo, Catarina Bravo, Alexandra M. M. Antunes, Vânia André

**Affiliations:** 1 Centro de Química Estrutural (CQE), Instituto Superior Técnico (IST), Universidade de Lisboa (UL), Av. Rovisco Pais 1, 1049-001 Lisboa, Portugal; paula.alves.marques@tecnico.ulisboa.pt (P.C.A.); catarinabravo@tecnico.ulisboa.pt (C.B.); alexandra.antunes@tecnico.ulisboa.pt (A.M.M.A.); 2 Associação do Instituto Superior Técnico para a Investigação e Desenvolvimento (IST-ID), Av. Rovisco Pais 1, 1049-003 Lisboa, Portugal; 3 Universidade Lusófona’s Research Center for Biosciences and Health Technologies (CBIOS), Campo Grande 376, 1749-024 Lisboa, Portugal; patricia.rijo@ulusofona.pt; 4 Research Institute for Medicines (iMed. ULisboa), Faculty of Pharmacy, Universidade de Lisboa (UL), Av. Prof. Gama Pinto, 1649-003 Lisboa, Portugal

**Keywords:** mechanochemistry, bioactivity, pipemidic acid, antibiotics

## Abstract

We report herein three novel complexes whose design was based on the approach that consists of combining commercially available antibiotics with metals to attain different physicochemical properties and promote antimicrobial activity. Thus, new isostructural three-dimensional (3D) hydrogen bonding frameworks of pipemidic acid with manganese (II), zinc (II) and calcium (II) have been synthesised by mechanochemistry and are stable under shelf conditions. Notably, the antimicrobial activity of the compounds is maintained or even increased; in particular, the activity of the complexes is augmented against *Escherichia coli*, a representative of Gram-negative bacteria that have emerged as a major concern in drug resistance. Moreover, the synthesised compounds display similar general toxicity (*Artemia salina* model) levels to the original antibiotic, pipemidic acid. The increased antibacterial activity of the synthesised compounds, together with their appropriate toxicity levels, are promising outcomes.

## 1. Introduction

Quinolone antibiotics are broad-spectrum synthetic antibacterial compounds that exhibit, in most cases, appropriate oral absorption and bioavailability [1,2]. This class of compounds has been reported to have a “nonclassical” biological impact, revealing antitumor, anticancer and antiviral activities [3,4,5]. Currently, there are four generations of quinolone antibiotics developed through the introduction of different structural changes on the typical quinolone skeleton. These modifications overcame some of the issues of the previous generations of these drugs, such as increased bacterial resistance and improvement of their physicochemical properties [6,7,8]. The activity of quinolones results from its interference with DNA synthesis and replication. In bacteria, these processes are performed by two topoisomerase type II enzymes: DNA gyrase and topoisomerase IV, which are the main targets in most Gram-negative and Gram-positive bacteria, respectively [8]. The typical oxo-cyclic oxygen and the carboxylic acid side chain of quinolones are known to be essential binding sites to bacterial topoisomerase type II enzymes. This interaction is mediated by a water-metal ion bridge allowing the anchoring of the quinolone to the acidic residues of the enzymes, thus preventing DNA replication [8,9,10,11].

**Figure 1:**
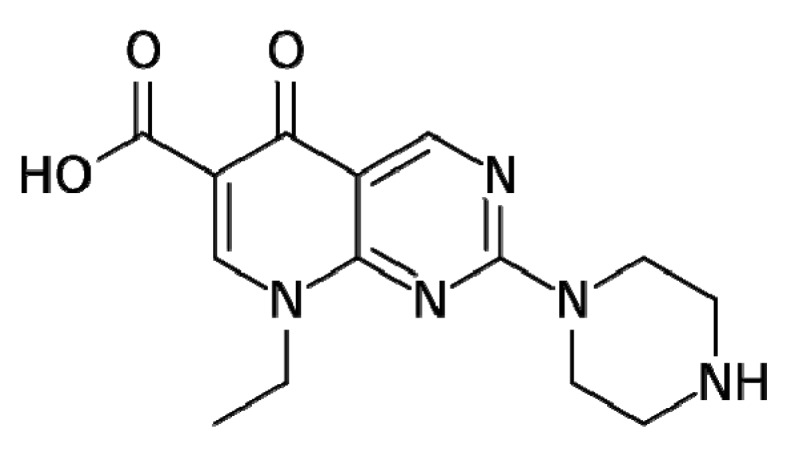
Pipemidic acid.

Pipemidic acid (Figure 1) was one of the first generation quinolone antibiotics to display enhanced activity due to the introduction of a piperazinyl side chain, while maintaining the aforementioned binding sites to the relevant enzymes [12,13]. This addition increased the lipophilicity of the compound, which has subsequently increased its ability to penetrate the bacterial cell wall [8,14]. Pipemidic acid is mostly used as a therapeutic agent to treat urinary tract infections due to its antibacterial activity against Gram-negative [15] and some Gram-positive bacteria [16,17].

However, this family of compounds has become ineffective at treating certain bacterial infections, despite the efforts to induce modifications to the typical quinolone structure once bacteria have been shown to rapidly adapt and develop resistance [6,9]. Bacterial resistance might be a result of mutations in bacterial genes (such as those encoding the quinolone target enzymes) or acquisition of resistance genes from other resistant strains [8]. Although the primary targets of quinolone compounds are known, the pathway that leads to cell death remains unclear, as for many other antibiotics.

The coordination of quinolones to biocompatible metals is of considerable interest from biological and pharmaceutical point of views and it has been shown to be an alternative approach to obtain compounds with improved antimicrobial activity while simultaneously altering their physicochemical characteristics [18,19]. Our group has proven that bio-inspired metal–organic frameworks of nalidixic acid, the first quinolone antibiotic, have increased solubility and bioactivity compared to the free antibiotic [20,21]. Efthimiadou et al. studied the antimicrobial activity of several pipemidic acid complexes, as well as their interaction with calf-thymus (CT) DNA with diverse spectroscopic techniques, and showed that the complexes are able to bind to DNA; in all the complexes, a B → A CT DNA transition is detected [22].

So far, several metal complexes with pipemidic acid have been reported [23,24]. Some enclose metal centres of cadmium [25], nickel [26], silver [27,28], zinc [29,30,31,32], manganese [30,33], cobalt [30] and copper [34,35,36,37]. However, to the best of our knowledge, few studies reporting their antimicrobial activity have been disclosed.

Bearing all this in mind, the selected biocompatible metals for this study were calcium, manganese and zinc. Each of these metals exhibit important roles in biological systems. Calcium is an abundant metal in the human body and it is vital in several cellular physiological and biochemical processes, such as signal transduction pathways [38], enzyme cofactor [39] and intercellular contacts [40]. Manganese is essential for bone strength but is also involved in enzymatic reactions such as the detoxification of superoxide free radicals in biological systems [21,41]. Zinc is an important trace metal in human nutrition that is crucial for many physiological functions and is involved in several enzyme actions in biological systems [42,43].

Herein, we present three isostructural hydrogen bonding frameworks built from pipemidic acid metal complexes successfully prepared by mechanochemistry [44,45,46], a privileged synthetic technique, in the solid state due to its reduced reaction times, lack or minimal usage of solvent, selectivity enhancement and novel reactivity [47,48]. Within the mechanochemistry in this study, a special emphasis has been given to liquid-assisted grinding (LAG), in which catalytic amounts of solvent are used [49]. The synthesised frameworks were structurally characterised by X-ray diffraction techniques and FT-IR spectroscopy and their shelf and temperature stability were also evaluated. The antimicrobial efficacy of the newly obtained compounds was tested against *Escherichia coli* and *Staphylococcus aureus*. Additionally, their general toxicity levels were also taken into account by a simple, fast and low-cost lethality bioassay using the brine shrimp *Artemia salina* [50,51].

## 2. Results and Discussion

**Figure 2:**
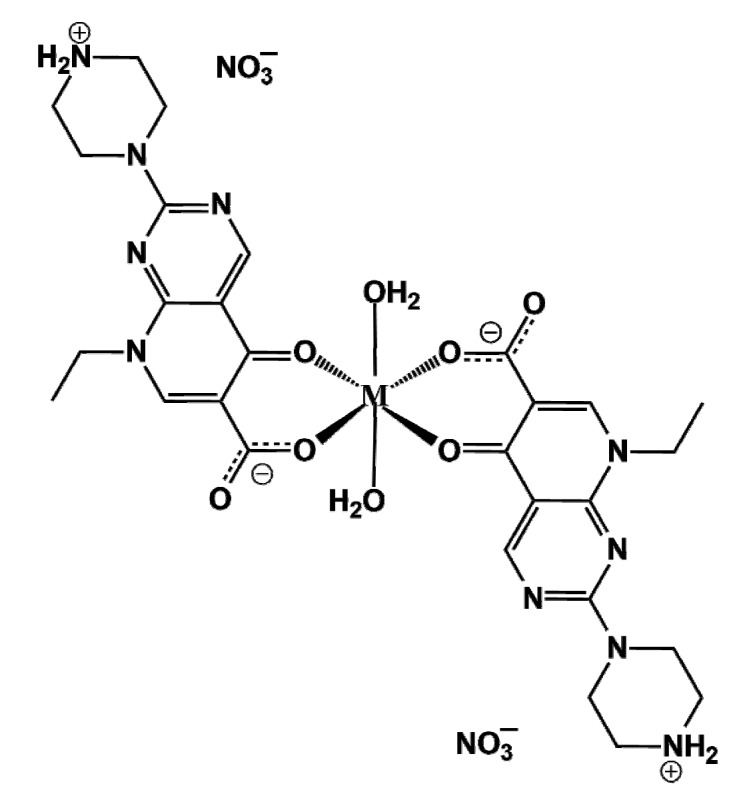
General representation of complexes **I–III**: (M(PA)_2_(H_2_O)_2_)(NO_3_)_2_, where M = Mn(II), Zn(II) and Ca(II), respectively, and PA = pipemidic acid.

Herein we unveil three novel isostructural pipemidic acid metal complexes with the generic formula [M(PA)_2_(H_2_O)_2_](NO_3_)_2_, where PA = pipemidic acid and M = Mn(II), Zn(II) and Ca(II) for compounds **I**, **II** and **III**, (Figure 2).

These complexes were synthesised by LAG and were obtained as pure phases in very high yields. Their structural characterisation is carefully discussed. Stability assays were performed, as well as tests to assess their general toxicity and antimicrobial effect against Gram-positive and Gram-negative bacteria.

### 2.1. Structural Characterisation

**Figure 3:**
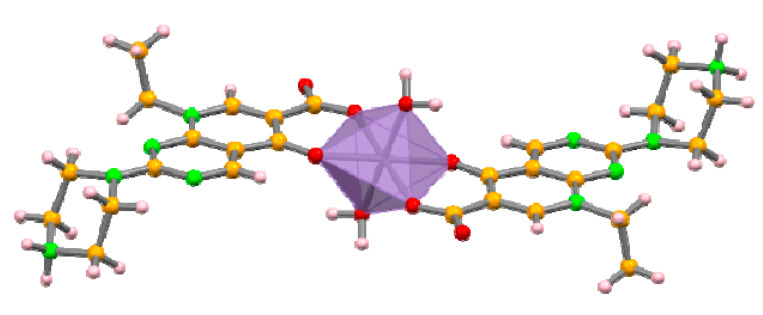
Crystal structure of complex **I**, depicting the distorted octahedral geometry of the metal centre, similar to complexes **II** and **III**.

The asymmetric unit of the complexes consists of a metal atom residing on an inversion centre coordinated to one zwitterionic pipemidic acid and a water molecule, with a non-coordinated nitrate anion ensuring the charge balance. The metal centre assumes a distorted octahedral geometry (Figure 3). Pipemidic acid coordinates to the metal in the equatorial positions via the carbonyl and carboxylate moieties. Water molecules assume the axial positions.

**Figure 4:**
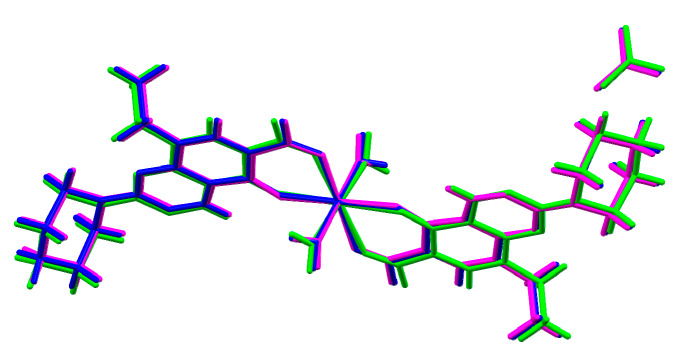
Overlap of the three isostructural complexes (**I** = blue; **II** = pink; **III** = green).

The overlap of the three structures (Figure 4) and the powder X-ray diffraction analysis (see Supplementary Materials Figure S1) clearly reveals the similarities of the crystal structures, with complex **III** presenting slightly higher deviations. These deviations are due to the slightly longer coordination distances to the metal centres (2.093(3), 2.181(3) and 2.205(4) Å for complex **I**, 2.015(3), 2.109(2) and 2.129(3) Å for complex **II** and 2.251(4), 2.322(3) and 2.353(4) Å for complex **III** (see Table S1 in Supporting Information for detailed bond distances and angles), which is most likely influenced by the larger atomic radii of calcium.

**Figure 5:**
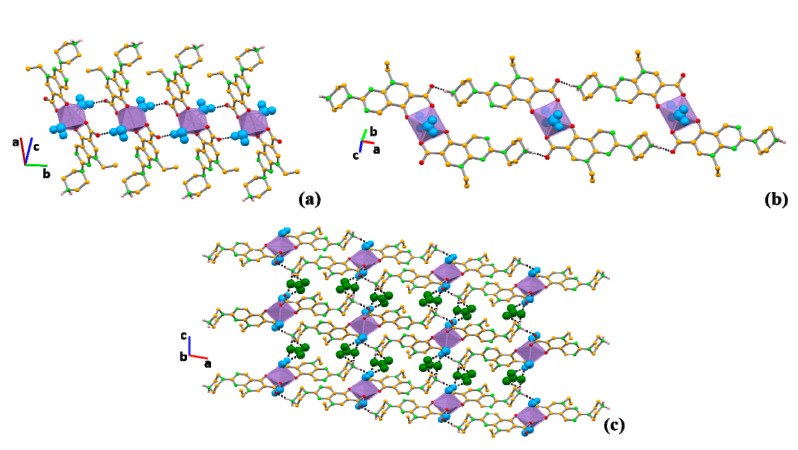
Supramolecular arrangements depicting (**a**) the hydrogen-bonded chains formed by the interaction between the water molecules and pipemidic acid; (**b**) the hydrogen bonds established between pipemidic acid in consecutive complexes; (**c**) the three-dimensional (3D) framework. Hydrogen atoms not involved in hydrogen bonds have been omitted for clarity; using spacefill style, water molecules are represented in blue and nitrate anions are represented in dark green.

Although complexes **I**–**III** do not form coordination frameworks, a careful look into their supramolecular arrangements reveals that three-dimensional (3D) frameworks are built based on hydrogen bonds, i.e., complexes **I**–**III** are hydrogen bonding frameworks (Table 1). Deconstructing these frameworks shows that the water molecules interact with the non-coordinated oxygen atom of the carboxylate moiety of pipemidic acid (O_1w_–H_1w_⋯O_2_), giving rise to chains that grow along the *b* axis (Figure 5a). The same oxygen atom involved in this interaction with the water further interacts with the NH_2_^+^ of pipemidic acid in a neighbouring complex (N_5_–H_2N_⋯O_2_, Figure 5b), creating a 2D framework in the *ab* plane. The nitrate bridges these 2D frameworks, acting as an acceptor for the water molecules (O_1w_–H_2w_⋯O_5_ and O_1w_–H_2w_⋯O_6_) and for the NH_2_^+^ of pipemidic acid (N_5_–H_1N_⋯O_4_ and N_5_–H_1N_⋯O_6_), assigning the three-dimensionality to the structures (Figure 5c).

**Figure 6:**
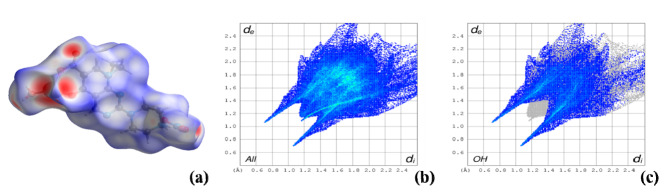
Hirshfeld surface analysis for complex **I** (**a**) and 2D fingerprint plots for the full interactions (**b**) and for the O-H interactions (**c**), similar results for complexes **II** and **III**.

The analysis of the Hirshfeld surfaces (Figure 6) and their associated two-dimensional fingerprint plots [52] complements the study on the packing interactions (Figures S2 and S3 in Supplementary Materials). As expected, the O–H interactions involved in the hydrogen bonds previously described are the most abundant (41.0%, 41.2% and 39.3% for complexes **I**, **II** and **III**, respectively), clearly identified as the red spots in the Hirshfeld surface and represented as the two spikes in the 2D fingerprint plots, corresponding to the lower distances. The second most relevant interactions in these structures are H–H contacts (29.1%, 30.3% and 29.1% for complexes **I**, **II** and **III**, respectively). The C–H interaction contributes approximately 8–9% (8.7%, 8.3% and 8.4% for complexes **I**, **II** and **III**, respectively) and N–H contributes 5.6%, 5.6% and 5.5% for complexes **I**, **II** and **III**, respectively. All the other interactions are in minor percentages (see Figures S2 and S3 in Supplementary Materials).

Infrared spectroscopy validates the crystal structure information, with the bands in the range of 3000–3600 cm^−1^ indicating the different hydrogen bonds in the complexes and the stretching vibration of water molecules that are coordinated to the metal centre (see Figure S4 in Supplementary Materials).

The structural characterisation of these novel compounds indicates that the typical quinolone oxo-cyclic oxygen and the carboxylic acid side chain, known as the binding sites to the bacterial topoisomerase type II enzymes, are involved in the coordination to the metal centre in these complexes.

#### Shelf and Thermal Stability of the Compounds

**Figure 7:**
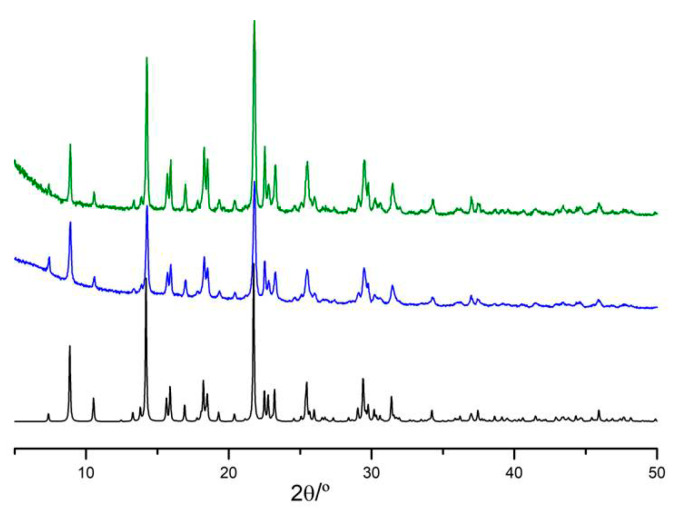
Powder X-ray diffractograms of complex **I** recorded after mechanochemistry (in blue) and after 5 months on a shelf (in green), at ambient condition, compared to the respective simulated diffractogram (in black).

The three compounds have been shown to be stable under shelf conditions (~50% room humidity and 20–25 °C) for at least five months. After five months, complex **III** undergoes some structural changes detected by powder X-ray diffraction at low 2θ angles, while complexes **I** and **II** remain stable (see Figure 7 and Figures S5–S7 in Supplementary Materials).

Regarding thermal stability, differential scanning calorimetry (DSC), thermogravimetric analysis (TGA) and hot-stage microscopy (HSM) data (see Figures S8–S11 and Table S2 in Supplementary Materials) indicate that the three complexes are stable until approximately 100 °C, the temperature at which the water molecules are released from the structures, corresponding to weight losses of 4.5–5%. Above 200 °C, melting/decomposition is observed for all three cases.

**Figure 8:**
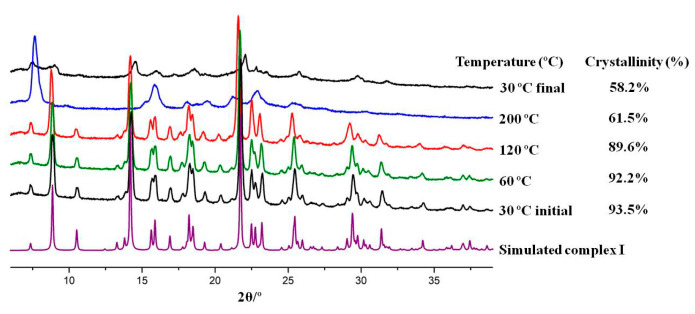
Variable temperature powder X-ray diffraction (VT-PXRD) of complex **I** recorded at five different temperatures and displaying different crystallinity patterns. The data obtained for complexes **II** and **III** is depicted in Figures S13 and S14 in Supporting Information.

Variable temperature powder X-ray diffraction (VT-PXRD) studies were conducted to further complement the thermal characterisation of these compounds. The three complexes undergo some structural changes and loss of crystallinity at temperatures above 120 °C (see Table S3 in Supplementary Materials). However, except for complex **I**, the pure initial form is obtained when returning to 30 °C and the crystallinity is recovered (see Figures S12–S14 in Supplementary Materials). For complex **I** (Figure 8), a different pattern corresponding to a form with low crystallinity (~58%) is detected when returning to room temperature.

### 2.2. Antibacterial Activity Assays

The synthesised complexes and corresponding starting materials were tested against *Escherichia coli* (Gram-negative) and *Staphylococcus aureus* (Gram-positive) bacteria to assess their antibacterial activity. Considering the minimum inhibitory concentration (MIC) values found for each compound (Table 2), it is clear that all the novel compounds are two-fold more active than the free antibiotic (pipemidic acid) against *E. coli*, an organism representative of Gram-negative bacteria and one major threat nowadays. Complex **III** displays differentiated activity against both strains, being more active against *E. coli*. With the exception of complex **III**, all compounds are more or equally effective against *S. aureus*. Among all, complex **I** seems the most promising compound, although its MIC value is similar to that of the respective metal nitrate salt. Finally, complex **II** presents similar MIC values for both strains; however, for *E. coli*, it is two-fold higher than pipemidic acid and for *S. aureus*, it is similar to the free antibiotic. NMR experiments performed in aqueous solutions indicate that the coordination of pipemidic acid on the complexes in this study is maintained. These results suggest that the enhanced activity for these complexes does not arise from a simple additive or synergistic effect between pipemidic acid and the cations (see Figure S15 in Supplementary Materials). This suggests that these complexes constitute promising alternatives to pipemidic acid, and their biological targets and/or mode of action should be further studied.

The minimum bactericidal concentrations (MBC) were also evaluated and, at the tested concentrations, all complexes and starting materials display a bacteriostatic effect on both *E. coli* and *S. aureus*.

### 2.3. General Toxicity Assay

**Figure 9:**
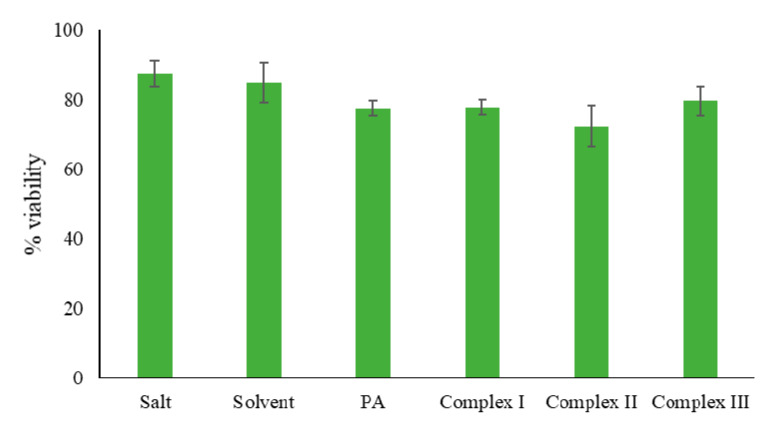
Graphical representation of the viability of *Artemia salina* after 24 h in the presence of 8 µg/mL of complexes **I**, **II** and **III**. PA = pipemidic acid. Sample solvent = DMSO (0.08% *v*/*v*).

The general toxicity of complexes **I**, **II** and **III** was assessed through a fast and simple lethality bioassay using the brine shrimp *Artemia salina* as a multicellular invertebrate model organism [50,51,53,54]. In order to get preliminary data, the complexes and the free antibiotic were tested at the concentration of 8 µg/mL, based on the MIC values found in the antimicrobial tests. Comparing the obtained results for all the synthesised complexes and pipemidic acid (Table 3 and Figure 9), it is possible to infer that they all have similar toxicity levels. Considering the increased antibacterial effect of these complexes, and their appropriate toxicity, these compounds appear to be very promising to pursue in further tests such as cellular viability assays.

## 3. Experimental Section

### 3.1. Reagents

The following reagents were used without further purification: calcium nitrate tetrahydrate (VWR International, Lisbon, Portugal), zinc nitrate hexahydrate (Scharlau, Barcelona, Spain), manganese nitrate tetrahydrate (Alfa Aesar, Kandel, Germany), ammonia 25% (Merck, Darmstadt, Germany), pipemidic acid (Sigma-Aldrich, Darmstadt, Germany) and DMSO (Fluka, Loughborough, UK).

### 3.2. Metal Complexes Synthesis

Complexes **I**–**III** were synthesised via manual mechanochemistry using 0.25 mmol of the metal salt and 0.50 mmol of pipemidic acid (Table 4) in the presence of catalytic amounts of water (100 μL) and ammonia (50 µL). Each metal salt (M) and pipemidic acid (PA) were manually ground for 5 min using a mortar and pestle in order to obtain complexes with a 1 M:2 PA ratio. Single crystals were obtained from recrystallisation of the resulting powder in water and left to crystallise by slow evaporation of the solvent at room temperature.

### 3.3. Single Crystal X-ray Diffraction (SCXRD)

Crystals of complexes **I**, **II** and **III** suitable for single crystal X-ray diffraction were mounted with Fomblin© in a cryoloop. Data was collected on a BRUKER D8 QUEST diffractometer with graphite-monochromated radiation (Mo Kα, λ = 0.7107 Å), at 293 K. The X-ray generator was operated at 50 kV and 30 mA, and the X-ray data collection was monitored by the APEX3 [55] program. All data were corrected for Lorentzian, polarization and absorption effects using SAINT + [56] and SADABS [57] programs. SHELXT [58] was used for structure solution and SHELXL-2014/7 [59] was used for full matrix least-squares refinement of *F*^2^. These three programs are included in the package of programs called WINGX version 2014.1 [60,61]. Non-hydrogen atoms were refined anisotropically. A full-matrix least-squares refinement was used for the non-hydrogen atoms with anisotropic thermal parameters. All hydrogen atoms bonded to carbon atoms were geometrically placed and refined in the parent carbon atom. Water hydrogen atoms and the hydrogen atoms connected to the nitrogen were located from the electron density map. MERCURY 4.3.1 [62] was used for packing diagrams and PLATON [63] for details on the hydrogen bond interactions. Crystal data and details of data collection and refinement for complexes **I**–**III** are reported in Table 5, and hydrogen bond details are given in Table 1. Crystallographic data of complexes **I** to **III** were deposited at the Cambridge Crystallographic Data Centre (CCDC 1956619-1956621).

### 3.4. Powder X-ray Diffraction (PXRD)

Powder X-ray diffraction data were collected on a D8 Advance Bruker AXS diffractometer with a Linxeye-XE detector and a copper radiation source (Cu Kα, λ = 1.5406 Å), operated at 40 kV and 30 mA, considering the 2θ range from 3–60°. The program MERCURY 4.3.1 [62] was used to obtain the diffraction patterns calculated from single-crystal data. The purity of the bulk was verified by comparing the calculated and observed powder X-ray diffraction patterns.

### 3.5. Variable Temperature Powder X-ray Diffraction (VT-PXRD)

The study of structural transitions with temperature the synthesised compounds was performed in a Bruker D8 ADVANCE powder diffractometer equipped with a SSD160 detector and a copper radiation source (Cu Kα, λ = 1.5406 Å), operated at 40 kV and 40 mA, considering the 2θ range from 6–40°. An Anton Paar HTK-16 high temperature chamber (RT-1400 °C) was coupled to this diffractometer. The diffractograms were acquired for five different specific temperatures for each compound during the total scan time of 171 s, with 300 s of delay for temperature stabilization, using a step size of 0.02°. The percentage of crystallinity was determined using the software DIFFRAC.EVA [64].

### 3.6. Hot-Stage Microscopy (HSM)

Hot-stage experiments were carried out using a Linkam TP94 device connected to a Linkam LTS350 platinum plate, using a 10 °C/min heating rate. The software Cell and an Olympus SZX10 stereomicroscope were used to collect the images.

### 3.7. Differential Scanning Calorimetry (DSC) and Thermogravimetric Analysis (TGA)

Combined measurements of samples (2–10 mg) were carried out on a SETARAM TG-DTA 92 thermobalance under nitrogen flow with a heating rate of 10 °C/min. Studies involving animals or humans require ethical approval must list the authority that provided approval and the corresponding ethical approval code.

### 3.8. Infrared Spectroscopy (IR)

Spectra of samples prepared in KBr (1:100 in weight) were recorded using a Nexus-Thermo Nicolet spectrometer (64 scans and resolution of 4 cm^−1^) in the 4000–400 cm^−1^ range.

### 3.9. Nuclear Magnetic Resonance Spectroscopy (NMR)

^1^H-NMR spectra were recorded on a Bruker Avance III 500 spectrometer, operating at 500 MHz.

### 3.10. Antibacterial Activity Assays

Gram-negative and Gram-positive bacteria (*Escherichia coli* (ATCC 25922) and *Staphylococcus aureus* (ATCC 25923), respectively) were selected as model organisms for the detection of the minimum inhibitory and bactericidal concentrations (MIC and MBC, respectively) of the complexes **I**–**III** and the respective reagents. These values were determined by the microdilution method [65]. Briefly, 100 μL of Mueller–Hinton liquid culture medium was added to all 96 wells of the microplate and 100 μL of the testing compounds at a concentration of 1 mg/mL were added to the first well. Serial dilutions of 1:2 were performed and 10 μL of bacterial inoculum added to each well. Then, the microplates were incubated for 24 h at 37 °C. After 24 h, the content of wells without active bacterial growth (within the range concentrations equal and higher to the MIC value) was used as inoculum to be spread onto a new sterile Mueller–Hinton solid culture medium. These solid medium plates were incubated at 37 °C for 24 h to allow MBC determination, which is the lowest concentration with no visible bacterial growth.

### 3.11. General Toxicity Assays

The synthesised complexes **I**, **II** and **III** were tested for their general toxicity through a fast and simple lethality bioassay using the brine shrimp *Artemia salina* as a multicellular invertebrate model organism [50,51,53,54]. Briefly, brine shrimp cysts obtained from JBL Artemia eggs (JBL GmbH and Co. KG, D-67141 Neuhofen Germany) were hatched in artificial seawater JBL Artemia salt (JBL GmbH and Co. KG, D-67141 Neuhofen, Germany) (i.e., salinity concentration of 30 g/L, following the indications of the manufacturer) and incubated under light for 48 h at 25 °C. On average, ten larvae were grown under the same conditions of light and temperature into 24-well culture plates (1 mL/well) containing a saline solution with 8 µg/mL of the compounds reported herein. This concentration value was chosen based on the minimum inhibitory concentrations found in the antimicrobial tests. After 24 h, the number of dead larvae was counted under a microscope and their viability was calculated using the following Equation (1):

(1) $$\%\;\mathrm{Viability}\;=\;\frac{\text{total number of larvae }-\text{number of dead larvae}}{\text{total number of larvae}}\;\times \;100$$

Independent duplicates were performed, each comprising four replicate groups of ten larvae per condition, and the results are expressed as percentage of the viability average ± standard deviation.

## 4. Conclusions

Herein we present three novel metal–coordination complexes of pipemidic acid with safe metals (Mn(II), Zn(II) and Ca(II)). Importantly, these compounds were synthesised from LAG mechanochemistry and their structural characterisation unveiled an isostructural character despite the different metal centres. These complexes are able to establish several strong hydrogen bonds, building 3D hydrogen bonding frameworks.

Further characterisation was carried out, envisaging possible pharmaceutical applications. The stability of these compounds to different environment conditions has shown that they are stable under shelf conditions (20–25 °C, ~50% RH), for at least five months, except for complex **III** which starts to undergo some structural changes after five months. Regarding temperature, they are stable until 100 °C. Compared to their parent antibiotic, all compounds display similar toxicity in *Artemia salina* and therefore, their effect should be tested in human cells. Notably, all the three complexes display bacteriostatic effect on both *S. aureus* and *E. coli*. Concerning the antibacterial activity, all the novel compounds presented increased activity when compared with pipemidic acid, with the exception of complex **III** against *S. aureus*. Complex **III** is more effective against *E. coli* than against *S. aureus*, while complex **I** seems more specific to *S. aureus*. Complex **II** displays a similar effect on both strains. Considering the escalating pressure to deal with the multi-resistance of *E. coli* strains, these complexes may be promising compounds to study further.

Overall, these results strongly advise the further comprehension of the mode of action and/or target of these complexes, as the typical binding sites with the bacteria enzymes are involved in the formation of the complexes. The bactericidal activity of quinolones has been reported as a result of their interference with the DNA synthesis pathway through a water–metal ion bridge, but new compounds can provide novel interaction sites or ways to interact. The successful isolation of these compounds not only enriches the realm of metalloquinolone complexes, but also provides new structural information that may help to understand the mechanisms of action of the quinolone antibacterial drugs. Ultimately, this work, along with additional studies, grants a possible strategy to battle the global threat of Gram-negative multi-resistant microorganisms.

## Figures and Tables

**Table 1:** Hydrogen bonding details for complexes **I**, **II** and **III**.

	Sym. Op.	D–H⋯A	*d* (D–H) (Å)	*d* (H⋯A) (Å)	*d* (D⋯A) (Å)	DĤA (°)
**I**	*x*, *y*, *z*	N_5_–H_1N_⋯O_4_	0.91(4)	2.43(5)	3.065(5)	126(4)
	*x*, *y*, *z*	N_5_–H_1N_⋯O_6_	0.91(4)	2.02(4)	2.936(6)	177(5)
	1 − *x*, 2 − *y*, 1− *z*	O_1w_–H_1w_⋯O_2_	0.90(3)	1.86(3)	2.742(4)	169(6)
	-½ + *x*, −½ + *y*, *z*	N_5_–H_2N_⋯O_2_	0.93(4)	1.82(4)	2.741(4)	170(4)
	½ + *x*, ½ − *y*, ½ + *z*	O_1w_–H_2w_⋯O_5_	0.89(6)	2.17(5)	2.990(6)	153(6)
	½ + *x*, ½ − *y*, ½ + *z*	O_1w_–H_2w_⋯O_6_	0.89(6)	2.22(6)	2.998(5)	146(5)
**II**	*x*, *y*, *z*	N_5_–H_1N_⋯O_4_	0.89(3)	2.58(3)	3.069(5)	116(3)
	*x*, *y*, *z*	N_5_–H_1N_⋯O_6_	0.89(3)	2.06(3)	2.933(5)	168(3)
	x, −1 + *y*, *z*	O_1w_–H_1w_⋯O_2_	0.88(2)	1.89(2)	2.753(3)	167(4)
	−½ + *x*, −½ + *y*, *z*	N_5_–H_2N_⋯O_2_	0.87(3)	1.86(3)	2.733(4)	174(4)
	½ − *x*, ½ + *y*, *z*	O_1w_–H_2w_⋯O_5_	0.87(3)	2.17(3)	2.974(5)	154(3)
	½ − *x*, ½ + *y*, *z*	O_1w_–H_2w_⋯O_6_	0.87(3)	2.27(3)	3.039(5)	149(3)
**III**	*x*, *y*, *z*	N_5_–H_1N_⋯O_4_	0.93(6)	2.55(7)	3.096(6)	118(5)
	*x*, *y*, *z*	N_5_–H_1N_⋯O_6_	0.93(6)	2.03(6)	2.952(6)	172(7)
	1 − *x*, 2 − *y*, 1 − *z*	O_1w_–H_1w_⋯O_2_	0.89(3)	1.85(4)	2.740(6)	172(7)
	½ + *x*, −½ + *y*, *z*	N_5_–H_2N_⋯O_2_	0.93(3)	1.86(4)	2.769(5)	168(5)
	−½ + *x*, ½ − *y*, −½ + *z*	O_1w_–H_2w_⋯O_5_	0.89(6)	2.43(7)	3.031(7)	125(6)
	−½ + *x*, ½ − *y*, −½ + *z*	O_1w_–H_2w_⋯O_6_	0.89(6)	2.09(6)	2.978(6)	171(5)

**Table 2:** Minimum inhibitory concentration (MIC) of each reagent and complexes **I**, **II** and **III** for *Escherichia coli* (Gram-negative) and *Staphylococcus aureus* (Gram-positive). The concentrations tested ranged from 500 to 0.061 µg/mL.

Compounds	*S. aureus*	*E. coli*
	MIC (µg/mL)	MIC (µg/mL)
Pipemidic acid	7.81	15.62
Mn(NO_3_)_2_·4H_2_O	3.90	62.50
Complex **I**	3.90	7.81
Zn(NO_3_)_2_·6H_2_O	31.25	31.25
Complex **II**	7.81	7.81
Ca(NO_3_)_2_·4H_2_O	7.81	62.50
Complex **III**	15.62	7.81
Negative control (DMSO)	62.50	62.50
Positive control	0.488 (VAN)	0.488 (NOR)

VAN = vancomycin; NOR = norfloxacin.

**Table 3:** Viability of *Artemia salina* after 24 h in the presence of 8 µg/mL of complexes **I**, **II** and **III**. After 24 h in the presence of 10% DMSO (% *v*/*v*, negative control), none of the *Artemia salina* in the respective well survived.

Sample	% Viability	% Viability Range
Salt	87.43 ± 3.77	83.66–91.20
Sample solvent	84.87 ± 5.70	79.17–90.57
PA	77.55 ± 2.00	75.55–79.55
Complex **I**	77.80 ± 2.09	75.71–79.89
Complex **II**	72.19 ± 5.93	66.26–78.12
Complex **III**	79.67 ± 4.07	75.60–83.74
Negative control	0 ± 0	0

Note: These results are expressed as percentage of the viability average ± standard deviation. PA = pipemidic acid. Sample solvent = DMSO (0.08% *v*/*v*).

**Table 4:** Masses weighed for the synthesis of complexes **I**–**III**.

Compound	Complex I	Complex II	Complex III
Pipemidic acid	0.1508 g	0.1519 g	0.1518 g
	(0.50 mmol)	(0.50 mmol)	(0.50 mmol)
M	0.0646 g	0.0778 g	0.0687 g
	(0.25 mmol)	(0.25 mmol)	(0.25 mmol)

Complex **I**: M = Mn(NO_3_)_2_·4H_2_O; Complex **II**: M = Zn(NO_3_)_2_·6H_2_O; Complex **III**: M = Ca(NO_3_)_2_·4H_2_O.

**Table 5:** Crystal data and structure refinement information for complexes **I**–**III**.

	I	II	III
Formula	(C_28_H_34_N_10_O_6_Mn·2(H_2_O))·2(NO_3_)	(C_28_H_34_N_10_O_6_Zn·2(H_2_O))·2(NO_3_)	(C_28_H_34_N_10_O_6_Ca·2(H_2_O))·2(NO_3_)
Fw	821.64	832.08	806.78
Crystal form, colour	Plate, colourless	Block, colourless	Needle, colourless
Crystal size (mm)	0.05 × 0.05 × 0.12	0.02 × 0.12 × 0.22	0.04 × 0.06 × 0.25
Crystal system	Monoclinic	Monoclinic	Monoclinic
Space group	*C*2/*c*	*C*2/*c*	*C*2/*c*
*a*, Å	24.347(5)	24.282(3)	24.533(7)
*b*, Å	6.9370(14)	6.8977(10)	7.0158(18)
*c*, Å	20.234(4)	20.316(3)	20.251(6)
*β* °	99.740(7)	100.656(5)	98.103(15)
*Z*	4	4	4
*V*, Å^3^	3368.1(12)	3344.1(8)	3450.8(16)
*D*_c_, g cm^−3^	1.620	1.653	1.553
*μ*(Mo K*α*), mm^−1^	0.482	0.825	0.270
θ range (°)	2.869–26.576	2.406–26.578	2.445–26.556
Refl. Collected/			
Independent refl.	25703/3513	16919/3410	57149/3561
*R*_int_	0.1696	0.1100	0.2113
*R*_1_^*a*^, *wR*_2_^*b*^ [*I* ≥ 2*σ*(*I*)]	0.0574, 0.1268	0.0628, 0.1605	0.0733, 0.1801
GOF on *F*^2^	1.039	1.017	1.103

*^a^ R*_1_ = Σ||*F*_o_| − |*F*_c_||/Σ|*F*_o_|. *^b^ wR*_2_ = [Σ(*w*(*F*_o_^2^ − *F*_c_^2^)^2^)/Σ(*w*(*F*_o_^2^)^2^)]^1/2^.

## Supplementary Materials

The following are available online, contains structural and FTIR data, Hirshfeld surface and 2D fingerprint plots analysis, details on shelf and thermal stability and ^1^H-NMR experiments.
