# Supplementary material for: Bioactivity of Isostructural Hydrogen Bonding Frameworks Built from Pipemidic Acid Metal Complexes
Source: Molecules. 2020 May 20;25(10):2374. doi: 10.3390/molecules25102374 (PMC7287797; doi:10.3390/molecules25102374)
Supplement: Supplementary file 1 [file molecules-25-02374-s001.pdf]

SUPPORTING INFORMATION

BIOACTIVITY OF ISOSTRUCTURAL HYDROGEN BONDING FRAMEWORKS BUILT FROM PIPEMIDIC ACID METAL COMPLEXES

Paula C. Alves[a,b], *Patrícia Rijo*[c,d], *Catarina Bravo*[a,b], *Alexandra M. M. Antunes*[a], *Vânia André*[a,b*]

[a] Centro de Química Estrutural, Instituto Superior Técnico, Universidade de Lisboa, Av. Rovisco Pais 1, 1049-001 Lisboa, Portugal; [b] Associação do Instituto Superior Técnico para a Investigação e Desenvolvimento (IST-ID), Av. Rovisco Pais 1, 1049-003 Lisboa, Portugal; [c] Universidade Lusófona's Research Center for Biosciences and Health Technologies (CBIOS), Campo Grande 376, 1749-024 Lisboa, Portugal; [d] Research Institute for Medicines (iMed. ULisboa), Faculty of Pharmacy, Universidade de Lisboa, Av. Prof. Gama Pinto, 1649-003 Lisboa, Portugal.

**vaniandre@tecnico.ulisboa.pt*

STRUCTURAL DATA

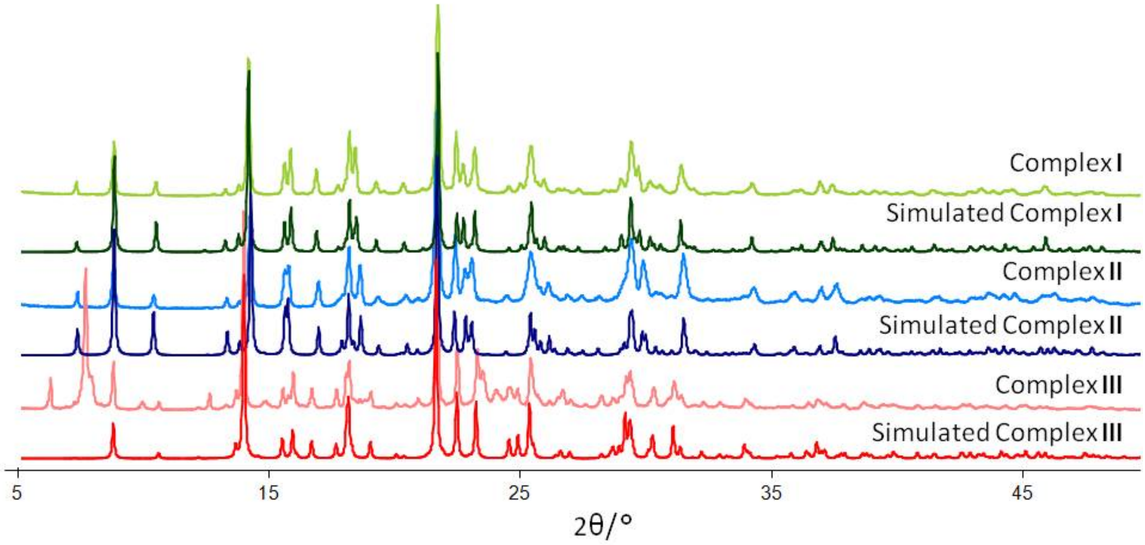

Figure S1. Powder X-ray diffractograms of complexes **I**, **II** and **III** recorded after mechanochemistry, at room conditions, and the respective theoretical diffractograms.

Table S1. Selected bond distances (Å) and angles (°) for complexes **I-III**.

	**I** (M=Mn)	**II** (M=Zn)	**III** (M=Ca)
M(1) - O(1)	2.093(3)	2.015(3)	2.251(4)
M(1) - O(3)	2.181(3)	2.109(2)	2.322(3)
M(1) - O(1W)	2.205(4)	2.129(3)	2.353(4)
O(1) - M(1) - O(1W)	92.82(12)	88.06(11)	95.40(14)
O(1) - M(1) - O(3)	83.02(10)	86.96(9)	76.67(12)
O(1) - M(1) - O(1)a	180.00	180.00	180.00
O(1) - M(1) - O(1W)a	87.18(12)	91.94(11)	84.60(14)
O(1) - M(1) - O(3)a	96.98(10)	93.04(9)	103.33(12)
O(1W) - M(1) - O(3)	88.04(12)	92.75(10)	89.78(14)
O(1W) - M(1) - O(1W)a	180.00	180.00	180.00
O(1W) - M(1) - O(3)a	91.96(12)	87.25(10)	90.22(14)
O(3) - M(1) - O(3)a	180.00	180.00	180.00

HIRSHFELD SURFACE AND 2D FINGERPRINT PLOTS

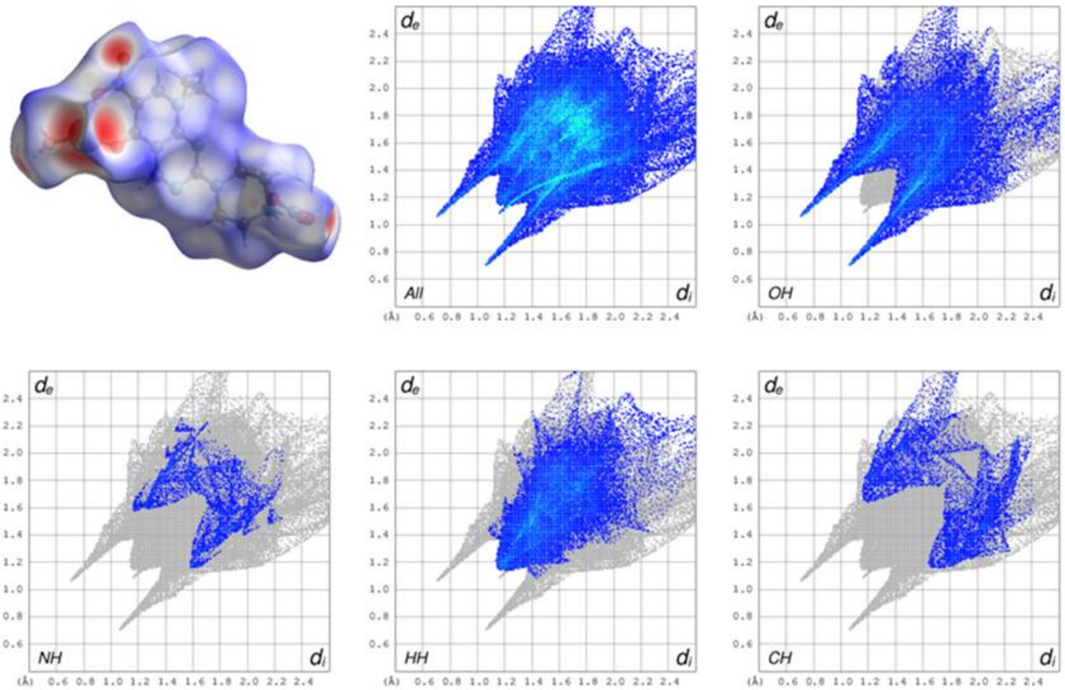

Figure S2. Hirshfeld surface and 2D fingerprint plots for complex **I**, similar to complexes **II** and **III**.

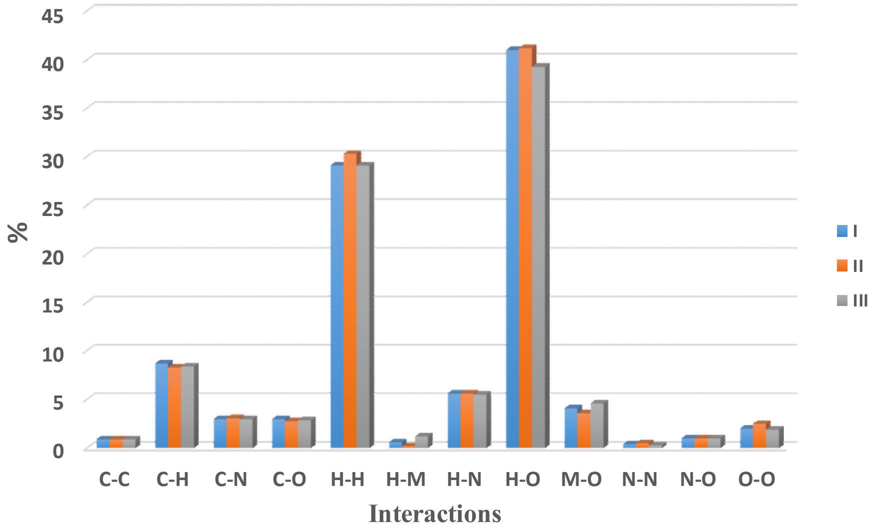

Figure S3. Summary of the percentage (%) of the interactions taken from the 2D fingerprint plots.

INFRARED SPECTROSCOPY DATA

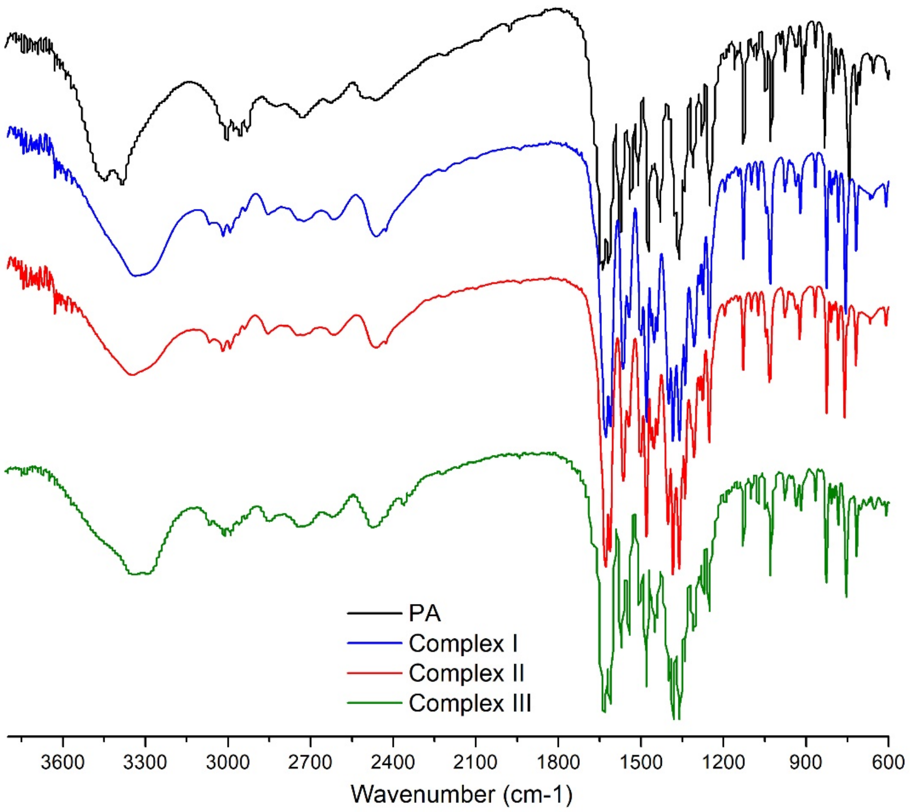

Figure S4. Fourier-transform infrared (FTIR) spectra of pipemidic acid (PA) and complexes **I-III** in KBr pellets.

SHELF STABILITY

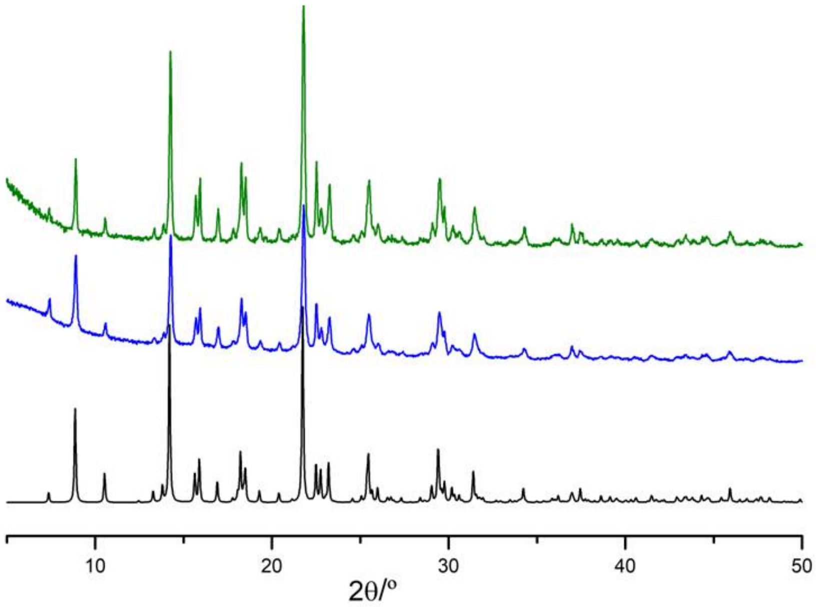

Figure S5. Powder X-ray diffractograms of complex **I** recorded after mechanochemistry (blue) and after 5 months on the shelf (green), at room conditions, compared to the respective simulated diffractogram from the solved crystal structure (black).

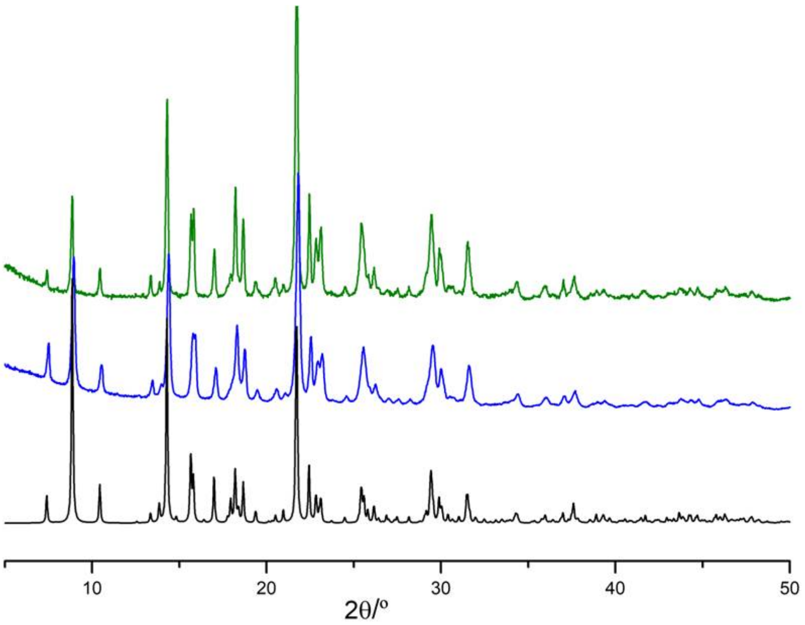

Figure S6. Powder X-ray diffractograms of complex **II** recorded after mechanochemistry (blue) and after 5 months on the shelf (green), at room conditions, compared to the respective simulated diffractogram from the solved crystal structure (black).

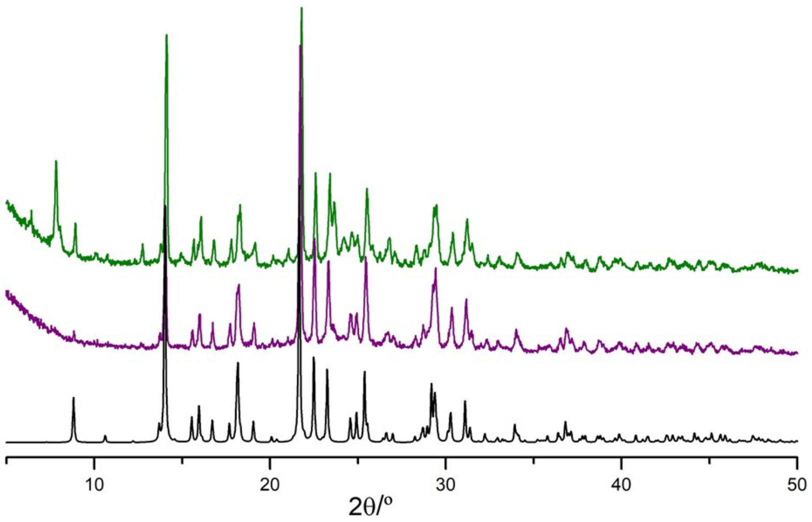

Figure S7. Powder X-ray diffractograms of complex **III** recorded after mechanochemistry (purple) and after 5 months on the shelf (green), at room conditions, compared to the respective simulated diffractogram from the solved crystal structure (black).

THERMAL STABILITY:

1) DSC/TGA DATA

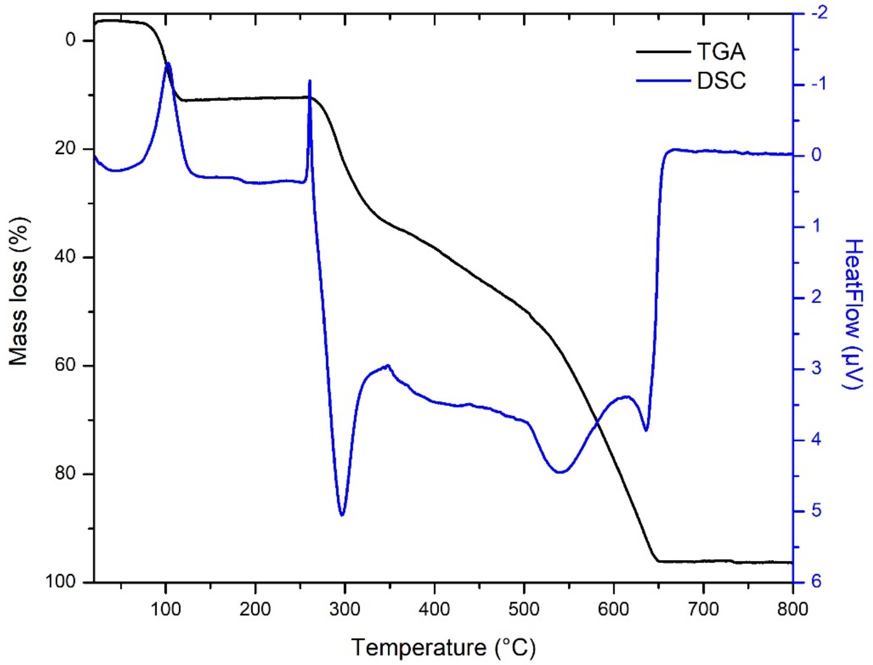

Figure S8. Thermogravimetry (TGA, black) and differential scanning calorimetry (DSC, blue) of pipemidic acid.

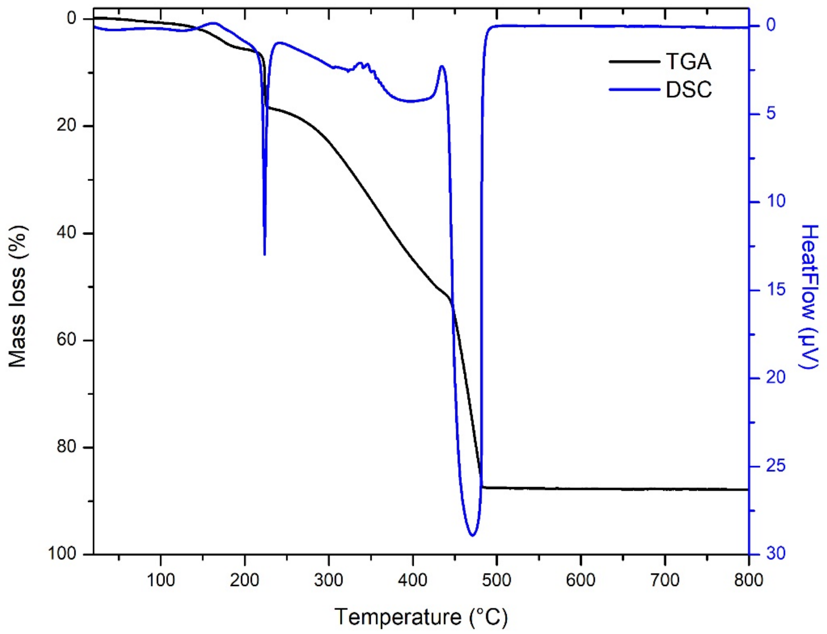

Figure S9. Thermogravimetry (TGA, black) and differential scanning calorimetry (DSC, blue) of complex **I**.

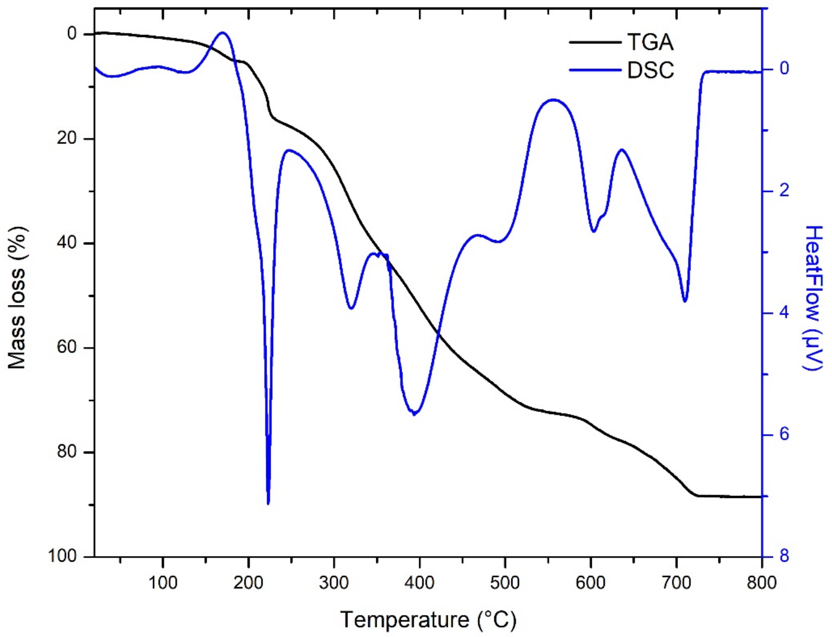

Figure S10. Thermogravimetry (TGA, black) and differential scanning calorimetry (DSC, blue) of complex **II**.

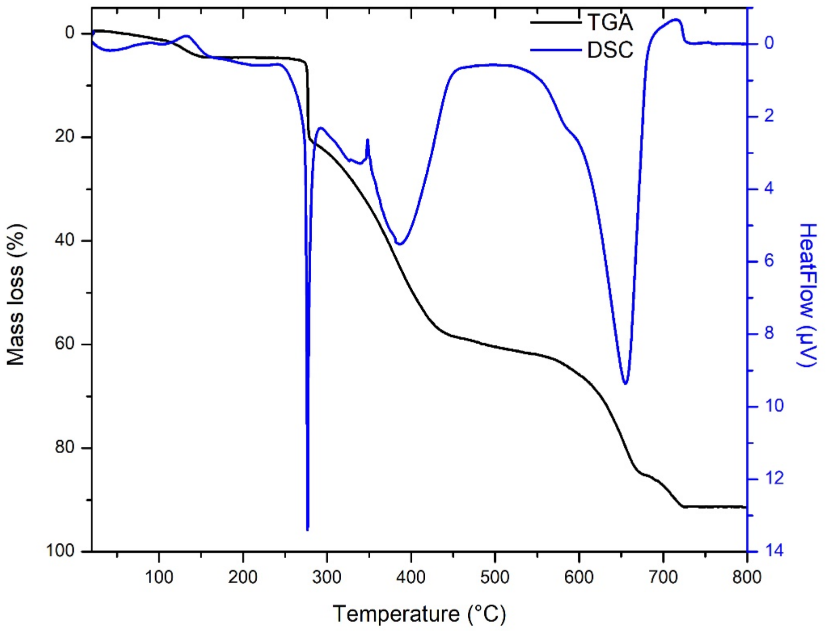

Figure S11. Thermogravimetry (TGA, black) and differential scanning calorimetry (DSC, blue) of complex **III**.

2) HOT-STAGE MICROSCOPY (HSM) DATA

Table S2. Images of hot-stage microscopy data for complexes **I**, **II** and **III**.

Complex I	Complex II	Complex III
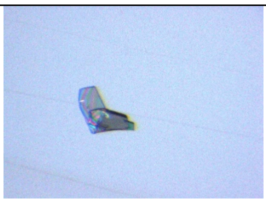 T=32°C	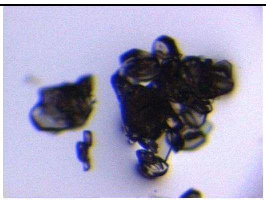 T=32°C	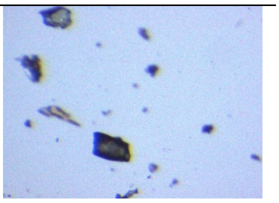 T=32°C
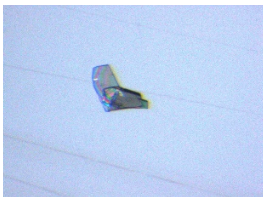 T=120°C	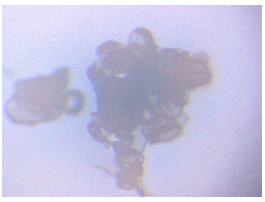 T=126°C	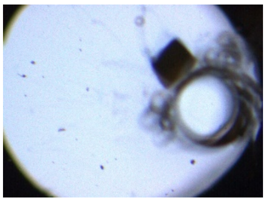 T=128°C
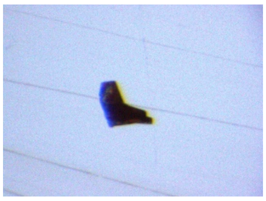 T=260°C	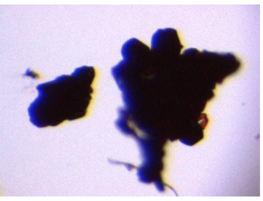 T=292°C	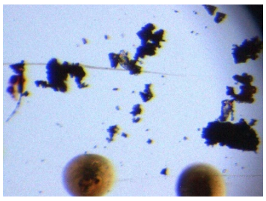 T=265°C

3) VARIABLE TEMPERATURE POWDER X-RAY DIFFRACTION (VT-PXRD) DATA

Table S3. Temperature variation of crystallinity of complexes **I**, **II** and **III**.

**Compound**	**Complex I**				
Temperature (°C)	30	60	120	200	30
Crystallinity (%)	93.5	92.2	89.6	61.5	58.2
**Compound**	**Complex II**				
Temperature (°C)	30	50	120	190	30
Crystallinity (%)	95.5	95.9	93.1	83.6	93.2
**Compound**	**Complex III**				
Temperature (°C)	30	50	80	160	30
Crystallinity (%)	94.4	93.8	94.3	80.0	93.2

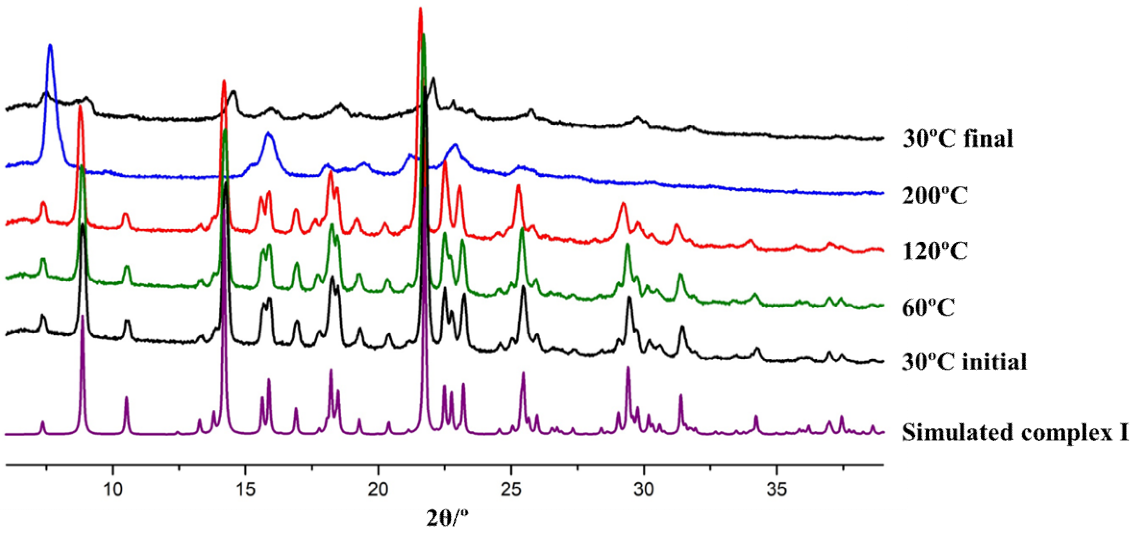

Figure S12. Variable temperature powder X-ray diffraction of complex **I** recorded at five different temperatures and displaying different crystallinity patterns.

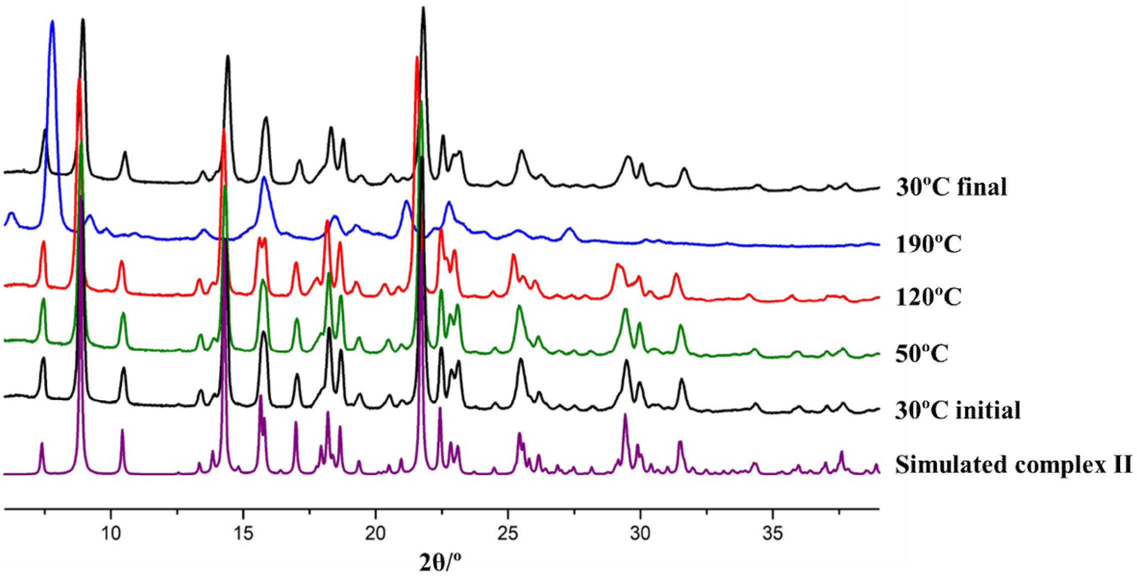

Figure S13. Variable temperature powder X-ray diffraction of complex **II** recorded at five different temperatures and displaying different crystallinity patterns.

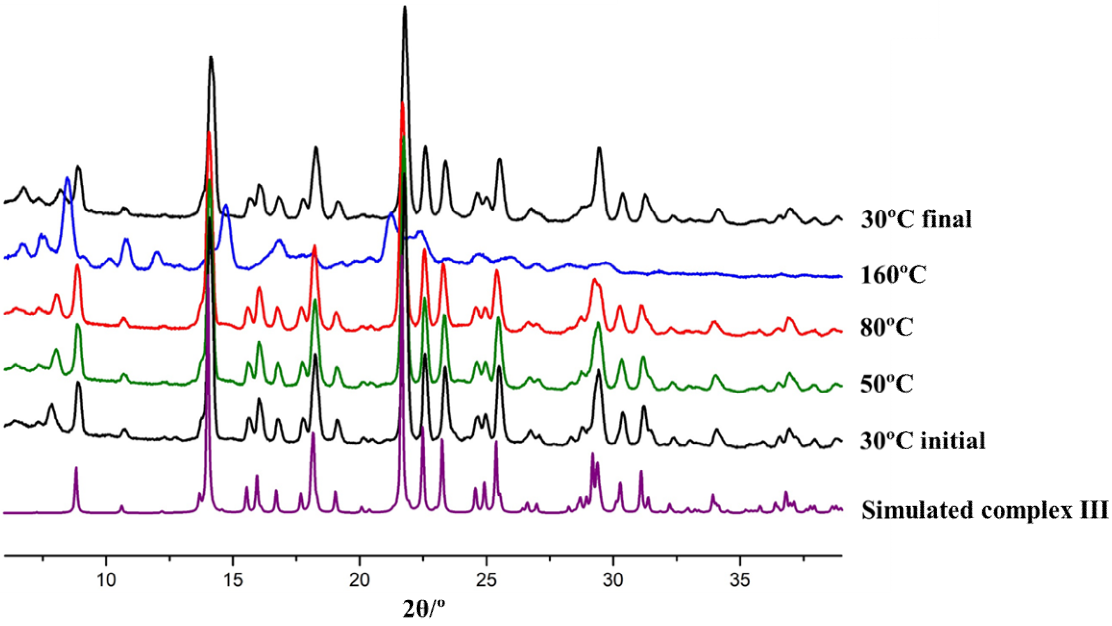

Figure S14. Variable temperature powder X-ray diffraction of complex **III** recorded at five different temperatures and displaying different crystallinity patterns.

NMR SPECTROSCOPY EXPERIMENTS

NMR spectroscopy experiments were performed to verify if the coordination of pipemidic acid on the complexes is maintained in aqueous solution. ^{1}H NMR spectra were obtained for complexes **II** and **III** and for free pipemidic acid in D_2O (**Figure S15**). The coordination can be confirmed by observable ^{1}H signal shifts of the coordinated pipemidic acid on the complexes compared to the spectra of the free antibiotic.

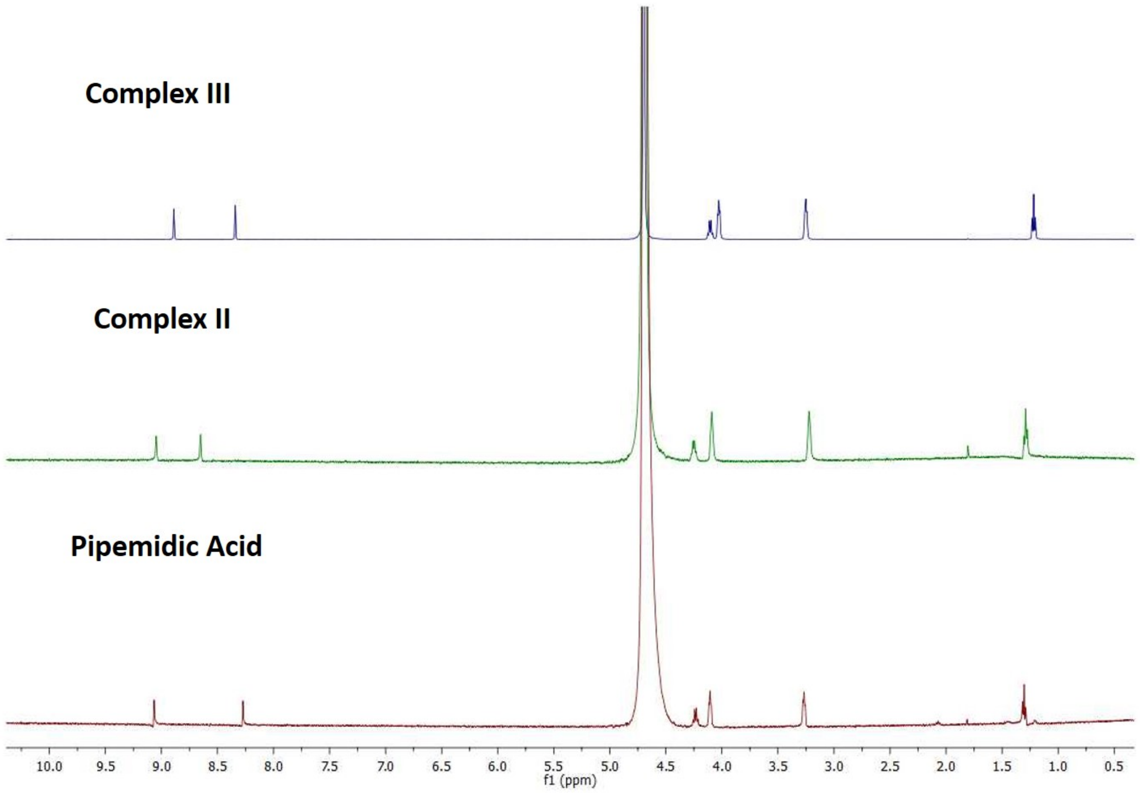

Figure S15. ^{1}H NMR spectra of the uncoordinated pipemidic acid, complexes **II** and **III** in D_2O.
